# Nanocurcumin-Based Sugar-Free Formulation: Development and Impact on Diabetes and Oxidative Stress Reduction

**DOI:** 10.3390/nano14131105

**Published:** 2024-06-27

**Authors:** Safa Ferradj, Madiha Melha Yahoum, Mounia Rebiha, Ikram Nabi, Selma Toumi, Sonia Lefnaoui, Amel Hadj-Ziane-Zafour, Nabil Touzout, Hichem Tahraoui, Adil Mihoub, Mahmoud F. Seleiman, Nawab Ali, Jie Zhang, Abdeltif Amrane

**Affiliations:** 1 Laboratory of Chemical Engineering, Chemical Engineering Department, Saad Dahlab University, Blida 09000, Algeria; ferradj_safa@univ-blida.dz (S.F.); nabi_ikram@univ-blida.dz (I.N.); hadj.ziane_amel@univ-blida.dz (A.H.-Z.-Z.); 2 Laboratory of Biomaterials and Transport Phenomena (LBMPT), Nouveau Pôle Urbain, Medea University, Medea 26000, Algeria; yahoum.madiha@univ-medea.dz (M.M.Y.); toumi.selma@univ-medea.dz (S.T.); lefnaoui.sonia@univ-medea.dz (S.L.); hichemm.tahraouii@gmail.com (H.T.); 3 LME, Material and Environmental Laboratory, University of Medea, Medea 26001, Algeria; 4 Functional Analysis of Chemical Processes Laboratory, Chemical Engineering Department, Saad Dahlab University, Blida 09000, Algeria; rebiha_mounia@univ-blida.dz; 5 Department of Nature and Life Sciences, Faculty of Sciences, Pole Urban Ouzera, University of Medea, Medea 26000, Algeria; nabil12marin.eco@gmail.com; 6 Laboratoire de Génie des Procédés Chimiques, Department of Process Engineering, University of Ferhat Abbas, Setif 19000, Algeria; 7 National High School of Chemistry of Rennes, Scientific Research National Center (CNRS), ISCR—UMR 6226, Rennes University, F-35000 Rennes, France; 8 Biophysical Environment Station, Center for Scientific and Technical Research on Arid Regions, Touggourt 30000, Algeria; adilmihoub15@yahoo.com; 9 Department of Plant Production, College of Food and Agriculture Sciences, King Saud University, P.O. Box 2460, Riyadh 11451, Saudi Arabia; mseleiman@ksu.edu.sa; 10 Department of Biosystems and Agricultural Engineering (BAE), College of Agriculture and Natural Resources, Michigan State University, East Lansing, MI 48824, USA; alinawab@msu.edu; 11 School of Engineering, Merz Court, Newcastle University, Newcastle upon Tyne NE1 7RU, UK; jie.zhang@newcastle.ac.uk

**Keywords:** nanocurcumin, biopolymers, encapsulation, *Stevia rebaudiana Bertoni*, antidiabetic activity, antioxidant activity, bioavailability

## Abstract

The objective of this study is the development of innovative nanocurcumin-based formulations designed for the treatment and prevention of oxidative stress and diabetes. Nanocurcumin was obtained through a micronization process and subsequently encapsulated within biopolymers derived from corn starch and fenugreek mucilage, achieving encapsulation rates of 75% and 85%, respectively. Subsequently, the encapsulated nanocurcumin was utilized in the formulation of sugar-free syrups based on *Stevia rebaudiana Bertoni*. The stability of the resulting formulations was assessed by monitoring particle size distribution and zeta potential over a 25-day period. Dynamic light scattering (DLS) revealed a particle size of 119.9 nm for the fenugreek mucilage-based syrup (CURF) and 117 nm for the corn starch-based syrup (CURA), with polydispersity indices PDIs of 0.509 and 0.495, respectively. The dissolution rates of the encapsulated nanocurcumin were significantly enhanced, showing a 67% improvement in CURA and a 70% enhancement in CURF compared with crude curcumin (12.82%). Both formulations demonstrated excellent antioxidant activity, as evidenced by polyphenol quantification using the 2.2-diphenyl 1-pycrilhydrazyl (DPPH) assay. In the evaluation of antidiabetic activity conducted on Wistar rats, a substantial reduction in fasting blood sugar levels from 392 to 187 mg/mL was observed. The antioxidant properties of CURF in reducing oxidative stress were clearly demonstrated by a macroscopic observation of the rats’ livers, including their color and appearance.

## 1. Introduction

Addressing the rising prevalence of diseases necessitates a focus on enhancing the nutritional quality of food. This can be achieved through the incorporation of nutrients known for their preventive or therapeutic properties, specifically targeting ailments commonly linked to unhealthy dietary choices and sedentary lifestyles [1]. Among the large number of phytochemical active ingredients, curcumin is a promising phytomolecule in the pharmaceutical field for its superior safety profile and affordability to all segments of the population [2]. Curcumin (CUR) is a naturally occurring polyphenolic compound and a potent hydrophobic, low molecular weight bioactive substance that is considered to be the main active agent in the plant turmeric (*Curcuma longa L.*), extracted from its rhizome [3]. Existing research data provide evidence to support the beneficial effects of curcumin on various human diseases, including joint inflammation, arthritis, diabetes, and other diseases [4,5,6,7,8]. Unfortunately, multiple drawbacks often limit the practical applications of curcumin, including its low water solubility and physicochemical instability, as well as its poor pharmacokinetics and low bioavailability rates [9]. Several researchers reported the development of multiple formulation methods to improve the solubility and bioavailability of curcumin, including emulsions [10], micro-emulsions [11], self-emulsifying systems [12], liposomes [13], and nanoparticle colloidal carriers [14]. Minimizing nutraceuticals to the nanoscale for in vivo delivery has shown highly promising outcomes and seems to be an advantageous approach in this regard [1]. The incorporation of curcumin into nanocarriers by various methods is an appropriate and successful choice to stimulate its biological activity, thus increasing its bioavailability, long-term circulation, and retention in the body and overcoming the physiological barriers of curcumin [6,15]. Nanocarriers based on biopolymers are considered the most favorable for the oral administration of curcumin because they are biocompatible and biodegradable. In this regard, natural polymer-based nanocarriers without sophisticated fabrication and chemical modification have been investigated for delivering curcumin [16]. Another approach for increasing the dissolution rate and absorption of curcumin is to increase its surface area by reducing the size of curcumin particles to the nanometric scale. The manufacture of nano-sized particles may be achieved by different methods, such as milling and grinding, and also by solvent-based processes like solvent evaporation or precipitation methods [17,18]. Ibrahim et al. (2019) reported that apart from nanocarriers, which are complex and difficult to implement, nanosizing also allows for improving the solubility and bioavailability of curcumin particles as well as their efficacy [19]. Kakran al. (2012) prepared nanosized curcumin, for oral administration via antisolvent precipitation as dry powder to overcome the barrier of the poor solubility in water and low bioavailability of native curcumin [20]. Basniwal et al. (2014) demonstrated the potential use of nanocurcumin aqueous dispersions as an adjuvant therapy for clinical application in treating various cancers [21]. Gera et al. (2015) also showed a much more effective bactericidal effect of the aqueous dispersion of nanocurcumin than normal curcumin [22,23]. Fenugreek mucilage (FG) mainly consists of galactomannan, which is extracted from the endosperm of Fenugreek (*Trigonella foenum-graceum*) seeds. FG is mainly composed of galactose, which is responsible for its high water solubility and highly viscous solutions [24,25]. FG is widely used in cosmetics and pharmaceuticals as a suspending agent because of the fact that it is non-toxic, nourishing for the skin, and very safe for oral use [26]. Starch is a very abundant polysaccharide in nature and is considered an interesting material for biomedical applications [27]. Starch is a carbohydrate stored in the stems and seeds of plants like rice and corn, which are its most important sources [28]. Corn starch (CS) is one of the most biocompatible compounds, mainly composed of highly branched amylopectin (α-d-(1-4) and α-d-(1-6)-glucosidic linkages) and amylose (linearly α-d-(1-4)-linked glucose residues) in a relatively constant ratio of 75:25 [29,30,31]. It is also used for obtaining nanoparticles as novel stabilizing agents for dispersions [27].

The main novelty of this work lies in the development of a sugar-free drinkable food supplement utilizing micronized curcumin. Micronization was employed specifically to enhance curcumin’s bioavailability and stability. The formulation cleverly incorporates natural excipients such as corn starch, fenugreek mucilage, and *Stevia rebaudiana*, which is the preferred non-calorie sweetener recommended by nutritionists, particularly in diets tailored for obese subjects and those dealing with diabetes [32], carefully chosen to impart organoleptic characteristics appealing to consumers. In vivo tests were conducted on Wistar rats to assess the formulations’ impact on type 2 diabetes and the reduction in oxidative stress. Additionally, in vitro tests were performed to determine antioxidant activity, highlighting the preventive potential against oxidative stress.

## 2. Materials and Methods

### 2.1. Materials

Curcumin (99%) was supplied by the BIOCHEM Chermopharma laboratory in France. *Stevia rebaudiana Bertoni* (95% of *Rebaudioside A*) was purchased from NATURA SAS, France. Corn starch (CS) was supplied by Sigma-Aldrich Laborchemikaliengmbh, and 2.2-diphenyl 1-pycrilhydrazyl (DPPH) was procured from PROCHEMA SIGMA, Germany. Streptozotocin (STZ) was obtained as a gift from Saidal CRD, Algeria. All other reagents used in this study were of analytical grade.

### 2.2. Micronization of Curcumin

Curcumin micronization was realized according to the method of [20], with some modifications. Curcumin (10 mg) was first dissolved in ethanol (5 mL) before being added dropwise to boiling water (50 mL) under homogenization for 1 h using an IKA T25 Ultra Turrax (Hambourg, Germany) at 8000 rpm.

### 2.3. Fenugreek Mucilage Extraction

*Fenugreek* mucilage was extracted from the seeds. First, the seeds were collected and washed with water to remove any impurities. Then, after they were macerated in distilled water at room temperature for 24 h, the *fenugreek* seeds were boiled for 1 h. After cooling, the filtrate was recovered by filtration and stored in a refrigerator overnight. Afterward, the filtrate was mixed with ethyl alcohol at a ratio of 1:1, and then the precipitated mucilage was filtered and dried at a temperature of 45 °C for 12 h in a laboratory oven (Memmert, UNB 400, Germany). The obtained product was ground and sieved (80 µm) to ensure homogeneous granulometry [33]. Finally, the extracted mucilage was subjected to an FTIR analysis before being used.

### 2.4. Formulation of Nanocurcumin Solutions

**Figure 1:**
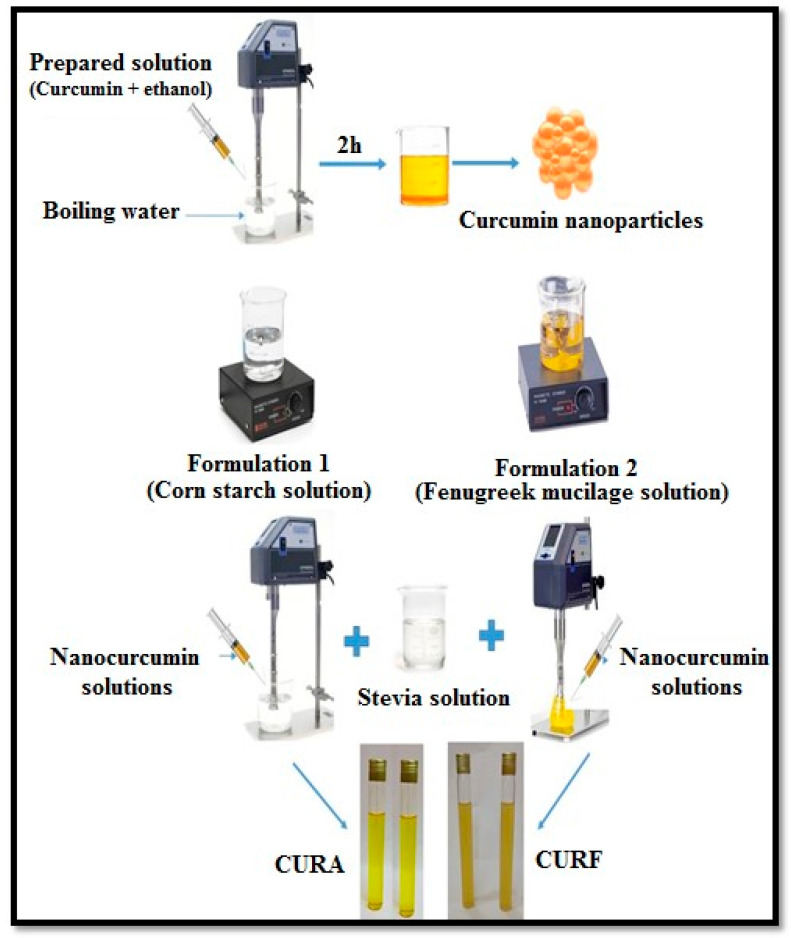
The development stages of nanocurcumin-based formulations (CURF and CURA).

The first step consisted of the preparation of encapsulating biopolymer solutions by heating 40 mL of distilled water, and then a quantity of 1 g of corn starch or *fenugreek* mucilage was added to the boiling water under stirring. In the second step, the nanocurcumin solution was added dropwise to the cornstarch or *fenugreek* mucilage solution with a syringe under homogenization. Afterward, a quantity of *rebaudioside A* was dissolved in cold water under stirring. Then, a volume of 10 mL of the *Bertoni Stevia rebaudiana* solution was added to the nanocurcumin solutions encapsulated in the biopolymers under homogenization, as described in Figure 1. Finally, the obtained formulations based on nanocurcumin encapsulated in corn starch (CURA) and *fenugreek* mucilage (CURF) were subjected to a series of characterizations.

### 2.5. Physico-Chemical Characterization of the Different Formulations

#### 2.5.1. Determination of the Particle Size and Zeta Potential of Nanocurcumin

The size and charges of the nanocurcumin particles were measured by dynamic light scattering (DLS) using a Horiba ZetaSizer (SZ-100-Z, Kyoto, Japan). Diluted samples in water (1%) were prepared and stabilized for 15 min under magnetic stirring before the data were collected as an average of three measurements. All measurements were collected at 25 °C with a detection angle of 90°.

#### 2.5.2. Fourier Transform Infrared Spectroscopy

FTIR analysis was used for the identification of the extracted *fenugreek* mucilage.

KBr pellets were first prepared by mixing 1 mg of the sample (fenugreek mucilage) with 99 mg of KBr. Then, the powdered mixture was ground evenly with a mortar before being compacted into a translucent disc under high pressure. The obtained sample disc was placed in an IR spectrophotometer (FTIR-8900 SHIMADZU, Kyoto, Japan), and the spectrum was recorded between 400 and 4000 cm^−1^ at a spectral resolution of 4.0 cm^−1^.

#### 2.5.3. Characterization of Nanocurcumin-Based Formulations

##### Physicochemical and Rheological Analysis

**(a)**  **The pH measurement:**

The pH was measured by the potentiometric method using a calibrated pH meter (HANNA, Cluj-Napoca, Romania). The mean pH value of three successive measurements was obtained by the direct immersion of the electrode into the sample.

**(b)**  **Density measurement:**

For the density measurement, a 10 mL pycnometer was used. Both the empty and filled pycnometers were weighed with a precision balance, and then the density of the product was deducted by calculation using the following formula (Equation (1)):

(1) $$d=\frac{m_{PS}-m_{PEmpt}}{m_{pw}-m_{PEmpt}}$$

where m_PS_ is the mass of the pycnometer with the sample, m_PW_ is the mass of the pycnometer with distilled water, and m_PEmpt_ is the mass of the empty pycnometer.

The measurements are carried out three times, and the results are averaged.

**(c)**  **Brix degree measurement:**

The Brix degree is used to identify the sugar content of a liquid. It was measured using a portable refractometer (MA871, Milwaukee, Romania). After homogenization, a few sample drops of the formulation were placed on the refractometer, and then the degree Brix value was determined at 25 °C. The measurements were repeated three times for each formulation.

**(d)**  **Determination of the particle size and zeta potential of the formulations:**

The particle size and zeta potential of the formulations were measured by DLS under the same operating conditions previously mentioned in Section 2.5.1.

**(e)**  **Study of rheological behavior:**

The rheological testing was carried out using an MCR 302 Anton PAAR PHYSICA rheometer (Graz, Austria) equipped with a cone and plate (60 mm) measuring device with a gap of 1 mm. The flow curves were determined at 20 ± 0.5 °C by the variation in the apparent viscosity (η_app_) as a function of the shear stress (γ^.^) in the range of 10^−3^ (s^−1^) to 10^3^ (s^−1^).

To determine the linear viscoelastic range for the CURA and CURF formulations, a strain sweep test was performed at a constant frequency (1 Hz) over a range of 0.1% to 100%.

##### Encapsulation Rate and In Vitro Dissolution Kinetics Study

For the determination of the nanocurcumin encapsulation rate, samples of 1 mL/100 mL were introduced into centrifugation tubes and then centrifuged at 3000 rpm for 10 min. Then, the supernatant from each tube was removed. The absorbance of both samples and formulations was measured using a UV–visible spectrophotometer (UV-1700 SHIMADZU, Kyoto, Japan) at 421 nm. The encapsulation rate of nanocurcumin was calculated by the Equation (2) below [16]:

(2) $$The\;rate\;of\;encapsulation\;(\%)=\frac{Abs\;of\;the\;sample}{Abs\;of\;the\;formulation}\times 100$$

where Abs is the absorbance.

A dissolution study was performed to compare the dissolution rate of the nanocurcumin formulations against the dissolution rate of crude curcumin in the intestinal simulated buffer (pH 6.8). The analysis was carried out in the dissolutest (ERWEKA DT 820, Germany). Initially, all dissolution vessels were filled with a 500 mL volume of the simulated intestinal fluid and then preheated to (37 ± 0.5) °C. Afterward, a formulation sample of 5 mL was introduced into each well under agitation at 100 rpm for 3 h continuously [34]. Then, aliquots of 5 mL were collected at the fixed time interval of 30 min using a syringe filter and replaced with an equal volume of fresh medium. Finally, the collected samples were analyzed using a UV–visible spectrophotometer at 421 nm.

##### Evaluation of the Biological Activities of the Formulations

The evaluation of the biological activities of the formulations focused on the determination of the in vitro antioxidant activity, on the one hand, and the in vivo anti-diabetic activity, on the other.

**(a)**  **In vitro antioxidant activity**

Antioxidant activity was determined for both formulations by the DPPH test. The protocol and reaction mechanism were described by [16]. A DPPH working solution was prepared by dissolving DPPH reagent in ethanol at a concentration of 0.1 mM. Then, to 4 mL of the above working solution, 1 mL of curcumin solution or blank aqueous solution was added. The mixture was shaken and left in the dark at room temperature for 30 min. Absorption at 421 nm was recorded on a UV-vis spectrophotometer. 

The DPPH radical-scavenging activity was estimated according to the following equation:

(3) $$DPPH\;radical\;scavenging\;activity\;(\%)=\frac{A_{blank}-A_{sample}}{A_{blank}}\times 100$$

where A_blank_ and A_sample_ are the absorption of DPPH solution added with distilled water and sample solution, respectively, at 421 nm. All the tests were performed in triplicate, and the results were expressed as means and standard deviation.

**(b)**  **In vitro hemocompatibility activity**

The ability of the formulations to produce hemolysis was assessed in accordance with a parameter to predict their hemocompatibility. In short, a 4:5 (*v*/*v*) dilution of whole blood from healthy individuals was performed using normal saline (0.9% NaCl). Accurate measurements of the formulas were taken. Each sample was incubated for 30 min at 37 °C after it was submerged in 5 mL of normal saline. The previously diluted blood was then added in the amount of 0.1 mL, and the mixture was incubated for an additional two hours at 37 °C. After centrifuging the samples for 10 min at 1500 rpm, the supernatant was extracted. A negative control was added to 5 mL of 0.9% NaCl with 0.1 mL of diluted blood. A positive control was also employed, which included 0.1 mL of diluted blood in 5 mL of double-distilled water. At λ = 545 nm, the absorbance was measured. The following formula was used to compute the hemolysis:

$$Hemolysis\;\%=\frac{Abs\left(sample\right)-Abs(negativecontrol)}{Abs\left(positivecontrol\right)-Abs(negativecontrol)}\times 100$$

All the protocols pertaining to the use of blood samples were approved by the Institute Ethics Committee, IIT Madras (IEC/2017/04/VMV/15).

**(c)**  **In vivo antidiabetic activity**

The aim of this study is to prove the efficacy of the nanocurcumin formulations on the regulation of blood sugar levels in rats made diabetic by induction with streptozotocin, a reference substance for experimental studies on diabetes [35].

Experimental animals: Twelve healthy adult male Wister strain rats (Pasteur Institute, Algiers, Algeria), 2 months old and weighing between 200 and 240 g, were used in this study. The rats were kept in plastic cages with stainless steel lids under standard laboratory conditions (room temperature, 12 h of light/12 h darkness). They were fed with standard pellets, sunflower seeds, and water ad libitum. The rats were divided into three groups of four rats per cage; each group was marked with different colored bands so that each rat could be clearly identified [35]. The distribution of each study batch was expressed as shown in Table 1.

Induction of diabetes: The dose of streptozotocin administered was 50 mg/kg by a single intraperitoneal injection into the left lower quarter of the abdomen of each rat in groups 2 and 3. The rats were then kept in cages with access to food and sweetened water (5% glucose solution) for 24 h to avoid hypoglycemic shock [36].

Each rat was immobilized in a restraint box before sanitizing the terminal end of its tail with a 20% ethyl alcohol solution. A drop of blood was then taken by a simple prick and deposited on a strip, which was then placed in a glucometer (Contour Plus, Basel, Switzerland) [37]. The pricked tail site was immediately sanitized to protect the rats from infection. Diabetes was confirmed after 10 days.

Intra-gastric gavage of rats with CURF: Group 3 rats were force-fed with a feeding tube and received a daily dose of 0.5 mL of fenugreek mucilage formulation (CURF) once a day on an empty stomach for 4 weeks of treatment [38,39]. Blood glucose was measured once a week.

**(d)**  **Pancreatic histology**

Following dissection, the pancreas was removed right away, washed in a cold physiological saline solution, and preserved in 10% neutral formalin diluted in 96% graded alcohol. Following that, the tissues underwent the treatment outlined by Perumal et al. [40]. The obtained serial sections were inspected with a (Leica DM500) optical microscope.

### 2.6. Statistical Analysis

The statistical analysis of all results was achieved via ANOVA using Tukey’s multiple comparison test. A *p*-value < 0.05 was considered statistically significant. All experiments were carried out in triplicate.

## 3. Results and Discussion

### 3.1. Determination of the Particle Size and Zeta Potential of Nanocurcumin

The particle size and polydispersity indices (PDIs) of the nanocurcumin were determined by DLS. The results obtained show, on the one hand, that the average particle size of the curcumin nanoparticles in CURA Formulation (1) is 102.8 nm ± 0.1 with a PDI of 0.390. On the other hand, the average particle size of the nanocurcumin in CURF Formulation (2) is 95.7 nm ± 0.1 with a PDI of 0.420, which confirms that the particle size of the nanocurcumin reduced (*p* < 0.05) from micrometer- to nanometer-sized. Furthermore, the PDI values are less than 1, indicating that the nanocurcumin solution size has a homogeneous particle size distribution pattern. Mostly, 10–100 nm-sized nanoparticles have been used for various medicinal applications and clinical trials [41].

**Figure 2:**
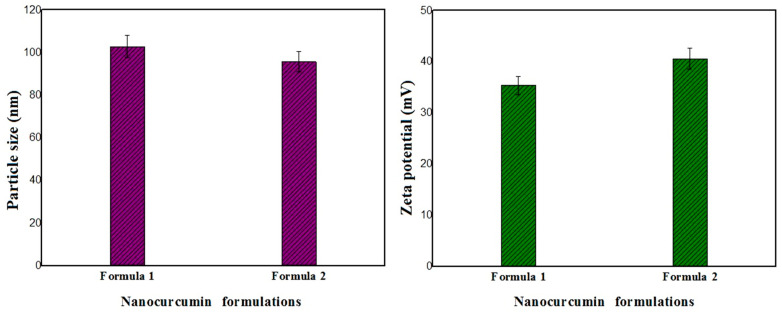
Histogram representing the variation in particle size and zeta potential of nanocurcumin formulations.

The zeta potential analysis shows that both curcumin nanoparticle formulations present positive zeta potential values of 35.4 mV ± 0.1 and 40.6 mV ± 0.1 for the CURA and CURF formulations, respectively (Figure 2). These results show the presence of strong repulsive electrostatic interactions among the curcumin nanoparticles. This helps prevent nanocurcumin particles from coming together and forming agglomerates, which contributes to the stability of curcumin nanoparticles.

### 3.2. Identification of Fenugreek Mucilage by Fourier Transform Infrared Spectroscopy (FTIR)

**Figure 3:**
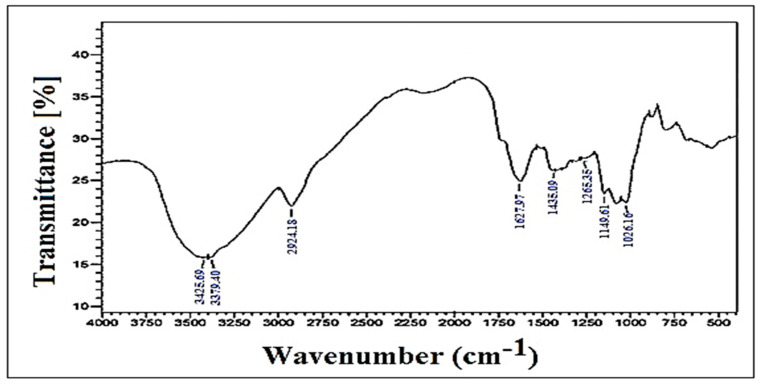
FTIR spectrum of fenugreek mucilage.

The FTIR spectrum of the fenugreek mucilage is illustrated in Figure 3, where the different peaks corresponding to the characteristic functional groups are clearly observed. The characteristic band between 3425.69 and 3379.40 cm^−1^ is attributed to the stretching of the hydroxyl (-OH) group. The peaks detected at 2924.18 cm^−1^ and 1627.97 cm^−1^ are characteristic of C-H and C-C bonds, respectively. A special observation is made for the two absorption bands between 1435.09 cm^−1^ and 1265.35 cm^−1^ and from 1149.61 cm^−1^ to 1026.16 cm^−1^ for the C-O group, with low intensity. 

The present results are in accordance with those reported by [33] for fenugreek mucilage isolated from *Trigonella foenumgraceum*.

### 3.3. Characterization of Nanocurcumin-Based Formulations

#### Physicochemical and Rheological Analysis

**(a)**  **pH measurement**

The pH testing results for both the corn starch-based syrup (CURA) and fenugreek mucilage-based formulation (CURF) are 6.22 ± 0.1 and 6.94 ± 0.1, respectively. These values are close to neutrality but remain acidic (<7). At acidic pH, curcumin stability is improved because of the fact that curcumin exists in its enolic form, which is more stable than the keto structure under alkaline conditions [42,43]. Evidenced curcumin degradation in the 7–10 pH interval led to maintaining the working pH below 7 to ensure stability [44].

**(b)**  **Density measurement**

The results of density measurements obtained show that the density of the fenugreek mucilage-based formulation CURF (1.15 ± 0.1) is slightly higher (*p* < 0.05) than that of corn starch-based syrup CURA (1.10 ± 0.1). This small difference is probably related to the nature and physico-chemical properties of the biopolymers used.

**(c)**  **Brix degree measurement**

Brix values provide information on the sweetness of products. The two formulations sweetened with *Stevia rebaudiana* showed a Brix level of 1.3341° for CURA and 1.3369° for CURF. These values are significantly lower compared with a sucrose-based solution (*p* < 0.05), where the Brix values are much higher [45]. Furthermore, no significant difference in Brix value was observed between the two formulations (*p* > 0.05). These results are very interesting for diabetic patients, knowing that stevia has a sweetening power 300 times higher than sucrose with zero calories [46], which makes it one of the most used sweeteners for weight control among obese individuals following a hypocalorie diet. It has also been reported that stevia decreased glucose blood levels and insulin resistance [46,47,48].

**(d)**  **Determination of the particle size and zeta potential of the formulations**

**Figure 4:**
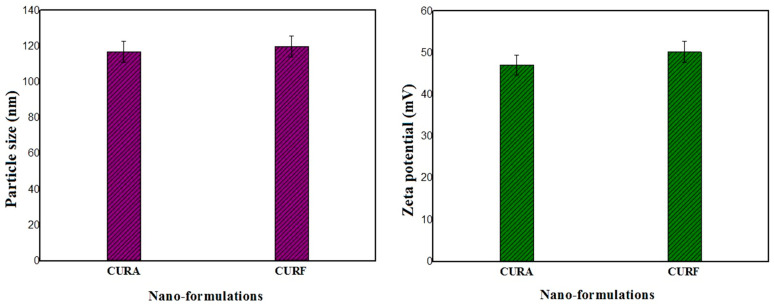
Histogram depicting the variation in particle size and zeta potential of the CURA and CURF formulations.

The particle size of encapsulated nanocurcumin in the formulated syrups is presented in Figure 4. The results obtained show that the average size of the two formulations, CURA and CURF, is 117 nm ± 0.1 and 119.9 nm ± 0.1, with polydispersity indices (PDI) of 0.495 and 0.509, respectively. These findings demonstrate that the encapsulation of the nanocurcumin in the corn starch and fenugreek mucilage biopolymers maintained a practically unchanged nanometric size of the nanocurcumin, allowing the formulations to be stable (*p* < 0.05). Otherwise, the particles of the unencapsulated nanocurcumin solution can aggregate, causing destabilization over time and an increase in particle size. Particle size, surface area, surface charge, and hydrophobicity are important physicochemical properties that make nanocurcumin more effective than native curcumin [49]. Characteristics of curcumin vary with particle size change on the nanoscale. It was previously found that particle size reduction considerably improves the effectiveness of nanocurcumin and makes it superior to native curcumin [41].

The zeta potential results obtained for the encapsulated curcumin nanoparticles in both CURA and CURF formulations show values of 47.1 ± 0.1 mV and 50.2 ± 0.1 mV, respectively. These values are slightly higher (*p* < 0.05) than those recorded for the non-encapsulated nanocurcumin solution.

Bhattacharjee (2016) previously reported that high-zeta potential nanoparticles are relatively stable because they repel each other and their collision frequency is non-existent or relatively low [50]. The electric potential of nanoparticles is defined by surface charge, and it is completely related to the chemical composition of nanoparticles.

Muller and Keck’s findings emphasize that both negative and positive zeta potentials play a crucial role in preventing the aggregation of nanoparticles. Curcumin tends to form aggregates and is susceptible to opsonization because of its limited solubility in water. Conversely, nanocurcumin demonstrates complete dissolution in aqueous media, avoiding the formation of aggregates [51].

The propensity of curcumin to aggregate and its susceptibility to opsonization are attributed to its low water solubility. In contrast, nanocurcumin’s distinctive ability to dissolve fully in aqueous environments eliminates the issue of aggregation [51]. In particular, nanoparticles with a positively charged surface are often considered advantageous, as this property facilitates enhanced penetration through cell membranes and leads to a higher absorption rate compared with negatively charged particles [52].

**(e)**  **Study of rheological behavior**

**Figure 5:**
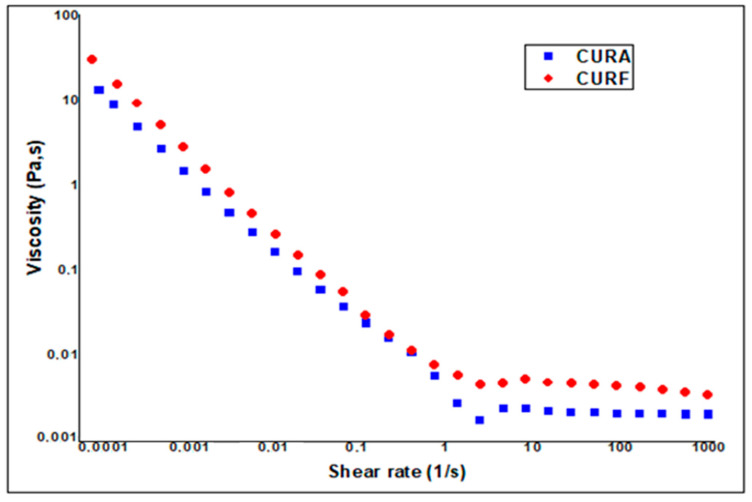
Evolution of the viscosity of the CURF and CURA formulations as a function of the shear rate.

Figure 5 shows the flow curves of the nanocurcumin syrup formulations under stress as a function of shear rate. The curves obtained show two different sections. The first is characteristic of non-Newtonian behavior, where it can be clearly seen that viscosity decreases with increasing shear rate (*p* < 0.05), corresponding to pseudoplastic and shear thinning behavior. The shear thinning behavior is related, on the one hand, to the disentanglement of polymer chains in the direction of flow and to the alignment of polymer chains in the direction of flow, which leads, on the other hand, to the lowering of polymer chain interaction [52,53].

On the other hand, the second section exhibits typically Newtonian behavior, where viscosity remains constant and invariant with increasing stress (*p* < 0.05). This could be explained by a fairly rigid structure at rest, which is immediately destroyed by the application of shear, resulting in a brittle network. As the shear rate increases, the polymer molecules and the randomly positioned chains become increasingly aligned in the direction of flow, resulting in linkage breakdown between adjacent polymer chains.

This behavior is perfectly suited to dilute solutions, such as our product. Nonetheless, it is observed that the fenugreek-based formulation (4 × 10^−3^ Pa·s) is slightly more viscous (*p* < 0.05) than the starch-based one (2 × 10^−3^ Pa·s). This demonstrates the higher rigidity of the fenugreek mucilage network, probably because of the presence of stronger interlacing (inter-intra) molecular interactions among the polymeric chains, which increases the stiffness of the gel network, making it more difficult to disrupt.

**Figure 6:**
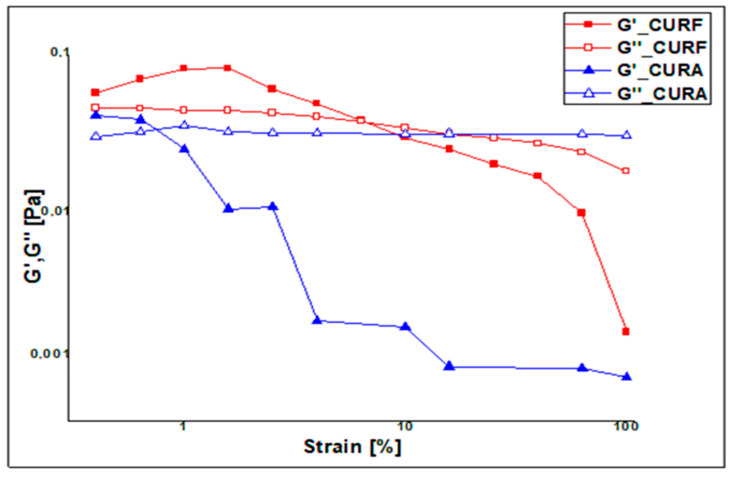
Viscoelasticity profiles of the CURA and CURF formulations.

Viscoelasticity tests confirm the results obtained under flow conditions. Figure 6 shows that in the linear domain, at low deformation, the behavior is slightly more elastic (*p* < 0.05) than viscous. This is reflected by G′ values that are slightly higher than G″ values, implying good stability of our product at rest.

The G′ of the samples was greater than G″ in the LVR region and before the flow point (G* corresponds to the cross point at which G′ = G″), which demonstrates solid-like (gel) behavior in the solutions (*p* < 0.05). However, it is observed that the fenugreek formulation presents a greater LVR range than the corn starch-based formulation (*p* < 0.05), again demonstrating the greater rigidity of the fenugreek mucilage polymer network, which indicates tougher interactions among the macromolecules and a more tightened structure [25,54].

When deformation exceeds the flow point (G*) value, the behavior reverses and becomes more liquid (G″ > G′). At high deformation, G′ values decrease more rapidly than G″, which, on the other hand, record a less rapid decrease or a plateau (*p* < 0.05) that corresponds to a rapid achievement of the Newtonian viscous liquid behavior by the products. However, it is clearly observed that the liquid behavior is more quickly established in the deformation range for the CURA formulation than in the CURF one, demonstrating once again the tighter structure of the fenugreek mucilage gel network [25,53,54,55,56].

### 3.4. Encapsulation Rate and In Vitro Dissolution Kinetics Study

The encapsulation rates of nanocurcumin in the formulations are 75% for CURA and 85% for CURF. Furthermore, the encapsulation rate of nanocurcumin in fenugreek mucilage is relatively better than that of corn starch (*p* < 0.05), which seems to be due to the characteristics of both polymers. These results are probably due, on the one hand, to the fact that the fenugreek mucilage forms a shell around the curcumin nanoparticles in the form of a transparent gel-like capsule, which represents a kind of modified cell wall with all the typical polysaccharides [57]. On the other hand, the outer surface of the starch is hydrophilic, but it has an open axial cavity, which is hydrophobic and capable of including other non-polar molecules in cases of geometric compatibility [58].

**Figure 7:**
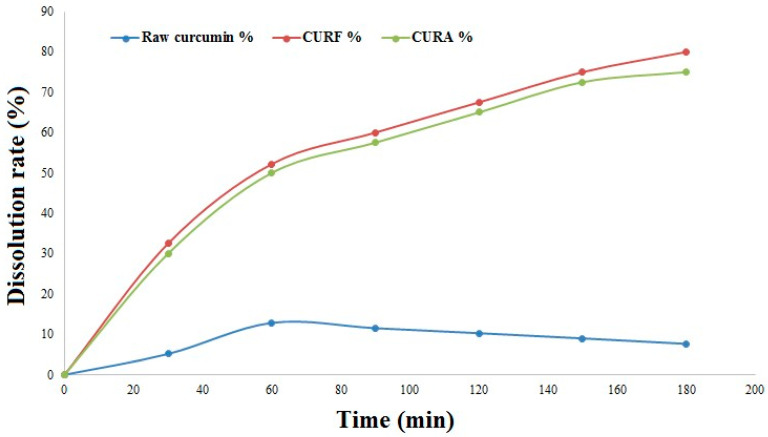
Dissolution profiles of crude curcumin and formulations.

The release profiles of nanocurcumin from the two syrup formulations, CURA and CURF, in addition to the dissolution profile of crude curcumin, in the simulated intestinal medium are shown in Figure 7. It is observed that the release rate of nanocurcumin encapsulated in corn starch (CURA) and fenugreek mucilage biopolymer (CURF) increases progressively with time to reach a maximum rate of 75% after 180 min and 82.5% after 210 min (indicating a controlled release of curcumin), respectively. Compared with crude curcumin, which reached a maximum rate of 12.82% after 60 min, the above results show that the dissolution rate was improved by 62% in CURA and 70% in CURF. Moreover, it is clearly noticed that the release rate of nanocurcumin encapsulated in fenugreek mucilage is relatively better compared with the release rate of nanocurcumin encapsulated in corn starch (*p* < 0.05). This may be due to the morphology of the encapsulation material and the mechanism of diffusion of the curcumin through the macromolecular network. The physiological mechanism of action is related to the water retention capacity through the hydrophilic matrix and the thickening properties of fenugreek mucilage, which cause a controlled slowing and release of the active ingredient [59]. For maize starch, the release of an active compound from a matrix-type delivery system can be controlled by diffusion, erosion, or a combination of both. Both homogeneous and heterogeneous erosion are detectable. Heterogeneous erosion occurs when degradation is confined to a thin layer on the surface of the delivery system, whereas homogeneous erosion is the result of degradation occurring at a uniform rate throughout the polymer matrix [60].

The surface area is also a paramount feature of nanoparticles. Primarily, materials made up of nanoparticles have a relatively larger surface area, which increases the rate of degradation and aqueous solubility and leads to increased bioavailability of drugs. Nevertheless, a large surface area enhances a drug’s response to a specific molecular target and improves its pharmacological activity [61].

The observed variations in dissolution rates between the two formulations and raw curcumin align with the existing literature findings [62]. These differences underscore the limited bioavailability of curcumin, a factor hindering its application in pharmaceuticals. This highlights the significance of downsizing curcumin to the nanoscale and encapsulating nanoparticles within biopolymers. This approach facilitates a controlled release mechanism for nanocurcumin, addressing the challenges associated with curcumin’s availability and enhancing its potential therapeutic efficacy.

### 3.5. Evaluation of Biological Activities

#### 3.5.1. Evaluation of Antioxidant Activity In Vitro

**Figure 8:**
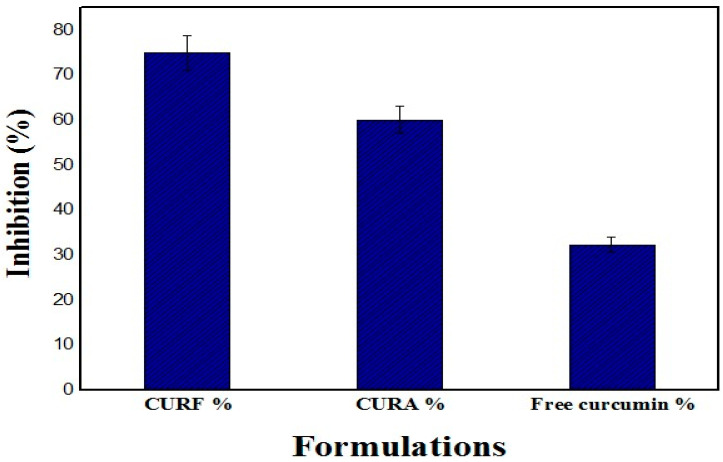
The percentage inhibition of different formulations.

The results of the antioxidant activity study demonstrated that the CURF formulation shows a higher percentage of inhibition, equal to 75%, compared with 60% for the CURA syrup (*p* < 0.05). It was also shown that the encapsulation of nanocurcumin in biopolymers considerably improves its free radical scavenging activity compared with free curcumin, which presents an inhibition percentage of 32.31% (Figure 8). This enhanced anti-oxidant activity in the two formulations compared with free curcumin may be due, on the one hand, to the presence of stevia in both formulations, as previously reported by [63,64].

On the other hand, the higher inhibition percentage of CURF is probably due to the additional antioxidant activity of the fenugreek mucilage, as demonstrated by [65], resulting from the synergistic effect of fenugreek mucilage with stevia and curcumin compared with CURA, which contains corn starch and is devoid of any antioxidant effect, as previously reported by [65,66,67].

#### 3.5.2. In Vitro Hemocompatibility Activity

In accordance with ISO 10993-4 [68], materials are deemed safe and hemocompatible if their hemolytic index is less than 5% [69].

**Figure 9:**
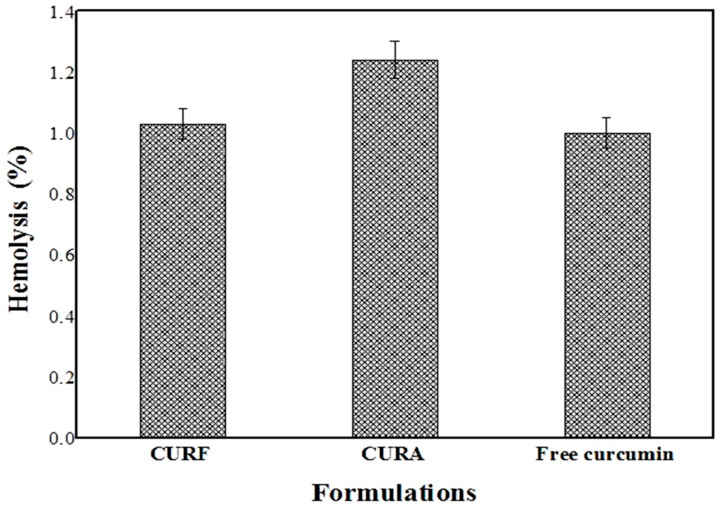
Hemolysis (%) of different formulations.

The acceptability of the formulations was verified by the evaluation of red blood cell hemolysis. Free curcumin, CURA, and CURF had hemolysis percentages of 1.00%, 1.24%, and 1.03%, in that order (Figure 9). The results demonstrated that the formulation components are biocompatible and can be used without causing acute hemolysis.

#### 3.5.3. In Vivo Antidiabetic Activity

According to all the results obtained, since the CURF formulation showed better physico-chemical and rheological characteristics in addition to a higher antioxidant activity compared with the CURA formulation, only CURF was retained for the test of anti-diabetic activity.

**(a)**  **Assessment of blood glucose levels:**

**Figure 10:**
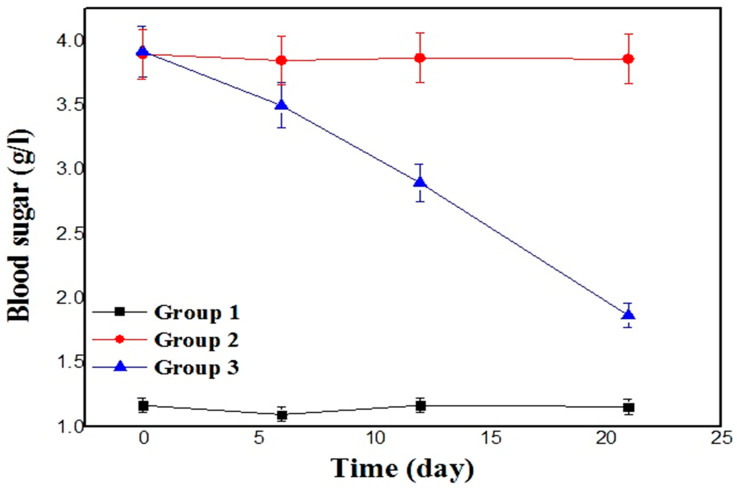
Fasting blood glucose levels of treated batches.

The results of the in vivo study on Wistar rats during a treatment period of 21 days are depicted in Figure 10. It was found that fasting blood glucose levels in group 1 (negative control) rats were below 120 mg/dL, confirming that the rats were healthy. For group 2 (positive control) rats, a single intraperitoneal injection of streptozotocin resulted in hyperglycemia (a diabetic state), with serum insulin levels lower than those in group 1 (negative control). Associated symptoms such as polydipsia, polyphagia, and polyuria, in which food and water intake were doubled compared to group 1 (the negative control), as well as weight loss because of excessive tissue protein breakdown, were also noted. The fasting blood glucose levels of rats in group 2 (positive control) were higher than those in group 1 (*p* < 0.05). Thus, all values were above 300 mg/dL, confirming the induction of diabetes by the administered dose of streptozotocin.

Group 3 was used to demonstrate the synergistic effect of the three compounds (curcumin, stevia, and fenugreek mucilage) in the CURF formulation, considering that the effects of each compound taken separately on the reduction in blood sugar have already been demonstrated and proven in previous works [70,71,72].

From the above curve, the gavage of group 3 rats with fenugreek mucilage formulation resulted in a decrease in food consumption, water, and urine production. In addition, body weight was limited compared with group 2 (positive control). On the other hand, a decrease in blood sugar levels from the seventh day of treatment, with a difference of 0.7 g, was observed. This improvement is due to the synergy of the three compounds (curcumin, stevia, and fenugreek mucilage) in the formulation compared with the effects of each compound alone and compared with the administered dose of the formulation [71,72,73,74]. The efficacy of the formulation on rats during the treatment period increased progressively until day 21, with a permanent reduction (*p* < 0.05) in blood glucose levels from 392 mg/mL to 187 mg/mL. Thus, the anti-diabetic effect of CURF syrup was significantly increased (*p* < 0.05). Fasting serum insulin levels, insulin sensitivity, and insulin resistance were significantly reduced. This was evidenced by a significant decrease in blood glucose levels with an improvement in β-cell function, leading to improved metabolism and transport of glucose to the cells, thus reducing oxidative stress [75,76,77].

Finally, the consumption of CURF allowed for the control of blood sugar levels and body weight. This last effect helps to prevent obesity in diabetic patients.

**Figure 11:**
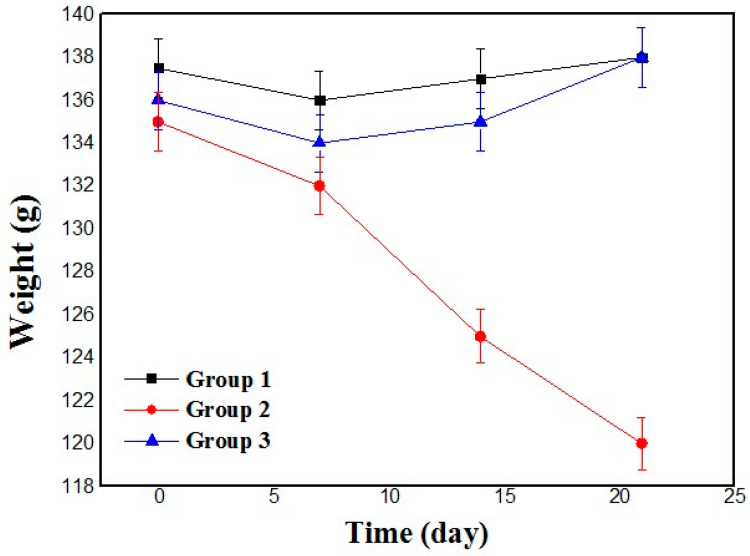
Rat weight evolution curves.

Figure 11 shows a significant reduction in the weight of the diabetic rats (group II) compared with their normal counterparts (*p* < 0.05). This decrease is attributed to the catabolism of structural proteins and lipids, which is thought to be a consequence of carbohydrate deprivation [74]. The observed weight loss in diabetic rats highlights the metabolic changes associated with the disease, particularly the breakdown of essential biomolecules because of the limited availability of carbohydrates. This information deepens the understanding of the physiological impact on weight dynamics in diabetic subjects and sheds light on the complex interplay between metabolic processes and dietary factors.

**(b)**  **Histological study**

**Figure 12:**
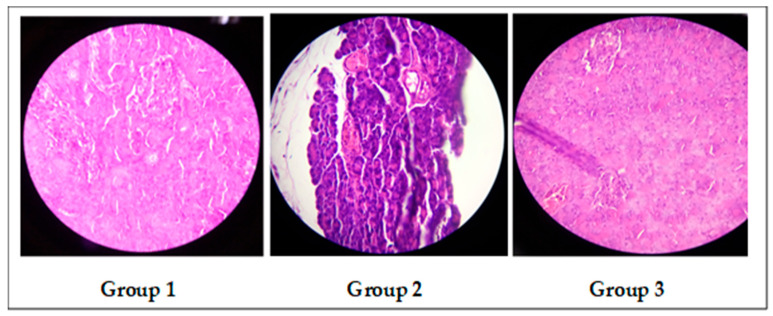
Histological microscopy of the pancreas.

Figure 12, above shows histological slices of a pancreatic representative of the experimental groups.

The pancreas of the rats in group 1 had an anatomical structure that displays regular Langerhans islets. Rats in the diabetic control group 2, on the other hand, had central neurosis and irregularly shaped islets with vacuoles. This pancreatic damage verifies the presence of diabetes and the efficacy of the chosen dosage of streptozotocin, which induces transient-type diabetes in rats [78]. However, the structures of the pancreas in the group 3 rats treated with our formulation showed no significant changes compared to the diabetic control group. This outcome emphasizes the need for the daily administration of the nanocurcumin-based formulation containing a variety of natural excipients. Thus, the ingestion of stevia, fenugreek mucilage, and nanocurcumin encouraged the regeneration of beta cells; the curcumin’s ability to defend against oxidative stress may be responsible for their restoration.

The usage of nanocurcumin exerted a more superior effect in the promotion of serum insulin levels, insulin pancreatic immunoreactivity, SOD, GSH, and PDX1 expression, and reduction in pancreatic caspase-3 than non-micronized CUR [79].

## 4. Conclusions

This present work led to the encapsulation of micronized nanocurcumin in fenugreek mucilage and corn starch using a simple solvent–antisolvent precipitation technique. The results obtained showed, on the one hand, that the reduction in the particle size made it possible to improve the physico-chemical properties and mainly the size of the curcumin particles from the micrometer to nanometer scale (in the order of 100 to 200 nm), as well as the surface area and the surface charge of the particles with zeta potential values higher than 30 mV. On the other hand, the used polysaccharides seem to have excellent protective properties for curcumin nanoparticles against environmental factors, degradation, and thus instability. Both the fenugreek-based (CURF) and corn starch-based (CURA) formulations offer a sustained release mechanism, with dissolution percentages of approximately 70% and 67%, respectively, in the physiological environment of 6.9 for 180 min. Because of its surface characteristics and optimal size, fenugreek mucilage has a high loading capacity to increase the therapeutic potential, pharmacological activities, and bioavailability of curcumin with a high rate of dissolution and encapsulation of nanocurcumin compared with crude curcumin, resulting in improved aqueous solubility and stability of the formulations. This study suggests that fenugreek mucilage has the best potential to be used as a carrier to release nanocurcumin and improve its aqueous solubility compared with corn starch. The in vitro tests demonstrated the synergistic antioxidant effect of the three compounds (curcumin, stevia, and fenugreek mucilage) compared with their individual antioxidant activities, which confirms the interest in the formulations for the prevention of oxidative stress. The monitoring of blood sugar levels for one month in rats made diabetic by streptozotocin induction also showed a remarkable hypoglycemic effect, as the fasting blood glucose levels were reduced from 392 to 187 mg/dL. The antioxidant activity study findings indicated that the CURF formulation exhibits a greater proportion of inhibition, specifically 75%, compared with 60% for the CURA syrup. Through pancreatic histological analysis, the cellular structures of the islets, specifically, the β cells, were restored along with the endocrine activity. In contrast, STZ caused significant degenerative changes that resulted in the islets shrinking in size and losing some of their cell count. Finally, the resulting fenugreek mucilage-based curcumin formulation represents an ideal candidate for the treatment and prevention of diabetes, particularly in prediabetic patients.

## Figures and Tables

**Table 1:** Batch allocation of the experimental study in Wistar rats.

Group	Designation
Group 1	Negative control (normal rats)
Group 2	Positive control (diabetic rats)
Group 3	Diabetic rats force-fed with fenugreek mucilage formulation

## Data Availability

The data presented in this study are available in the manuscript.
